# Mechanisms of change of a novel weight loss programme provided by a third sector organisation: a qualitative interview study

**DOI:** 10.1186/s12889-016-3063-4

**Published:** 2016-05-10

**Authors:** Naoimh E. McMahon, Shelina Visram, Louise A. Connell

**Affiliations:** College of Health and Wellbeing, University of Central Lancashire, Preston, PR1 2HE UK; School of Medicine, Pharmacy and Health, Durham University Queen’s Campus, Stockton-on-Tees, TS17 6BH UK

**Keywords:** Obesity, Overweight, Intervention, Evaluation, Mechanisms

## Abstract

**Background:**

There is a need for theory-driven studies that explore the underlying mechanisms of change of complex weight loss programmes. Such studies will contribute to the existing evidence-base on how these programmes work and thus inform the future development and evaluation of tailored, effective interventions to tackle overweight and obesity. This study explored the mechanisms by which a novel weight loss programme triggered change amongst participants. The programme, delivered by a third sector organisation, addressed both diet and physical activity. Over a 26 week period participants engaged in three weekly sessions (education and exercise in a large group, exercise in a small group and a one-to-one education and exercise session). Novel aspects included the intensity and duration of the programme, a competitive selection process, milestone physical challenges (e.g. working up to a 5 K and 10 K walk/run during the programme), alumni support (face-to-face and online) and family attendance at exercise sessions.

**Methods:**

Data were collected through interviews with programme providers (*n* = 2) and focus groups with participants (*n* = 12). Discussions were audio-recorded, transcribed and analysed using NVivo10. Published behaviour change frameworks and behaviour change technique taxonomies were used to guide the coding process.

**Results:**

Clients’ interactions with components of the weight loss programme brought about a change in their commitment, knowledge, beliefs about capabilities and social and environmental contexts. Intervention components that generated these changes included the competitive selection process, group and online support, family involvement and overcoming milestone challenges over the 26 week programme. The mechanisms by which these components triggered change differed between participants.

**Conclusions:**

There is an urgent need to establish robust interventions that can support people who are overweight and obese to achieve a healthy weight and maintain this change. Third sector organisations may be a feasible alternative to private and public sector weight loss programmes. We have presented findings from one example of a novel community-based weight loss programme and identified how the programme components resulted in change amongst the participants. Further research is needed to robustly test the effectiveness, and cost-effectiveness, of this programme.

**Electronic supplementary material:**

The online version of this article (doi:10.1186/s12889-016-3063-4) contains supplementary material, which is available to authorized users.

## Background

Overweight and obesity have reached epidemic proportions globally [1, 2]. In 2014 39 % of adults worldwide were overweight and 13 % were obese [3]. In the UK, it is expected that over half of the population could be obese by 2050 [4], costing the National Health Service (NHS) £10 billion per year [5]. People that are overweight or obese are at a higher risk of developing a range of chronic conditions including type 2 diabetes, hypertension, cardiovascular disease (including stroke) and cancer [6]. For many people weight loss is difficult to achieve. However, even modest losses of 5 to 10 % of body weight are associated with significant health improvements [7]. Dietary and physical activity interventions can result in significant and clinically meaningful weight loss [8], but this is often poorly maintained in the time period following the intervention [9].

It is now increasingly recognised that effective interventions are those that address *both* diet and physical activity, with the National Institute of Health and Care Excellence (NICE) identifying “multicomponent interventions” as the treatment of choice [10]. A systematic review of reviews by Greaves and colleagues [11] investigated the components of dietary and physical activity interventions that were associated with increased effectiveness. More effective interventions were found to be those that used established behaviour change techniques and those that encouraged participants to engage sources of social support, for example, families, friends and colleagues, in their planned behaviour change. Despite an improved understanding of the effective components of interventions, there still exists a paucity of evidence to support particular weight loss interventions in the UK. A randomised controlled trial by Jolly and colleagues explored the effectiveness of eight different interventions [12]. These included commercial weight loss programmes such as Weight Watchers, Slimming World, Rosemary Conley and NHS weight loss programmes. Commercial programmes were shown to be more effective than the primary care based NHS services which were deemed to be ‘ineffective’. More recently we have seen the emergence of bespoke weight loss programmes such as the Football Fans in Training (FFIT) programme [13]. FFIT is a male only weight loss programme delivered by community coaching staff at Scottish Premier League football clubs over 12 weekly sessions. When tested in a randomised controlled trial (RCT) this programme was found to be effective in helping men to lose a clinically important amount of weight.

For these types of complex interventions it is not sufficient to know whether or not an intervention works; we must also develop an understanding of how an intervention may work and for whom. This idea of exploring the underlying mechanisms of how a programme changes participants’ reasoning to bring about a set of outcomes is not a new phenomenon [14]. It has been a longstanding concern that assumptions about how programmes are intended to work are often not made explicit, thus compromising evaluation efforts [15]. Equally, if a programme has been built on faulty theory, then it may never bring about the anticipated outcomes regardless of the quality of implementation [16]. There is a need for theory-driven studies that explore the mechanisms of change of complex weight loss programmes. The aim of this paper is to present findings from a qualitative study exploring the mechanisms by which a novel weight loss programme, provided by a third sector organisation, triggered change amongst participants. Third sector organisations are non-governmental and non-profit-making organizations such as charities, voluntary and community groups and cooperatives. These organisations play an important role in health and social care services and may provide a worthwhile alternative for delivering weight loss programmes.

## Methods

### Overview of the intervention

The weight loss programme under investigation was Aspire. Aspire was born out of a need to address levels of obesity that are significantly higher than the national average in a particular borough in the North West of England. The rationale for the programme was that individuals who are very overweight or obese require a level of support, above and beyond that currently provided by NHS and commercial weight loss programmes, to make sustainable changes in both their dietary intake and physical activity patterns. The programme is delivered by two members of staff; both are qualified fitness instructors and one is a registered dietician. Each Aspire cohort is made up of 12 individuals and, at the time of writing, the programme is entering its fifth cohort. The programme has been funded through a Big Lottery Grant awarded to the third sector organisation. The effectiveness of the programme has not yet been formally evaluated. However, pre-post measures collected during the previous cohorts suggests that participants have been successful at losing significant amounts of weight. The content of the programme was not grounded in formal theory during development. It has evolved, and been refined, based on the experiences of the providers and client feedback after each cohort.

There are three sessions a week over the 26 week period, comprised of: (i) a full group session where half the time is devoted to physical activity and the other half to group discussion, (ii) a small group session where clients meet in their training groups (three groups of four) for physical activity and (iii) a one-to-one session between clients and the provider. There are only two criteria to be eligible for selection on to the Aspire programme. Clients must demonstrate that they can commit the time to attend programme sessions that take place during working hours and in the evenings, and they must not have complex medical problems which would be beyond the scope of the providers to comfortably manage (e.g. recent heart attack, complex/chronic musculoskeletal problems). Clients are assigned to one of the two providers at the outset and work with the same provider for the duration of the programme. They are allocated to the training groups based on gender and availability to attend sessions at different times. Full group sessions are held at the community gym, while the small group sessions and one-to-one sessions are held in various locations depending on the activities to be undertaken that day (e.g. gym or park). Clients are given free membership for the community gyms for the duration and for an additional six months after they have completed the programme. An overview of the Aspire schedule, including the content of the weekly sessions, is provided in Additional file 1: Aspire schedule.

### Study design and setting

We used a qualitative approach for this study, collecting data through focus groups and semi-structured interviews to explore participants’ views and experiences in depth. We have used the COREQ checklist in reporting the methods used in our study (Additional file 2: COREQ checklist). Focus groups and interviews were carried out in the first month after the completion of the 26 week programme at the local community centre where it was delivered. This is also the workplace of the programme providers.

### Sample

All clients of the fourth Aspire cohort (*n* = 12) and the two Aspire providers were invited to participate in the study. The lead researcher emailed all potential participants with an information sheet explaining the purpose of the study and what would be required. All participants provided written informed consent prior to taking part in the study. Aspire clients consented to (i) participating in a focus group, and (ii) allowing the research team to access data routinely collected by the Aspire providers (e.g. weight, body mass index (BMI), health and wellbeing measures). The rationale for collecting this data was to provide detailed descriptions of the participants, and the extent of their weight change after participating in the programme.

### Data collection

Focus groups were chosen as the data collection method for clients as they expressed that they would like to be interviewed in the groups in which they train during the programme. Discussions were audio-recorded and were led by NM using a topic guide developed from previous evaluations of weight loss programmes (Additional file 3: Topic guides). Only the interviewer (NM) and the participants were present during the focus group discussions. Routinely collected data were made available to the lead researcher in spreadsheet format by the Aspire providers.

Semi-structured interviews were conducted with the providers using an interview guide developed from behaviour change theory and intervention development literature (Additional file 3: Topic guides). The interviews were conducted by the lead researcher (NM) and audio-recorded. The Aspire providers were interviewed separately to explore their experiences of developing the weight loss programme, along with their perceptions of how the intervention components supported participants to make positive lifestyle changes. Topic guides were not piloted in advance of data collection. Minor changes were made to the ordering of questions following the first focus group and interview to facilitate improved flow of discussion. Focus groups and interviews lasted approximately one hour and additional field notes were not made. At the end of data collection no new themes were emerging and data saturation had been achieved.

### Data analysis

The focus groups and interviews were transcribed verbatim and imported into NVivo10 for content analysis. Transcripts were read a number of times for understanding. The lead researcher (NM) first free coded all transcripts to inform the development of an initial coding frame. To facilitate clarity and consistency in terminology, recently published behaviour change frameworks and taxonomies were used in the coding process. These included the Theoretical Domains Framework [17] to code determinants of target behaviours, the Behaviour Change Wheel [18] to code text on the functions of the weight loss programme and the Behaviour Change Technique Taxonomy (v1) [19] to code specific techniques used in individual programme components. New codes were created to capture text that did not fit within existing domains of each of the frameworks. This approach to coding allowed us to develop a better understanding of how the programme was intended to trigger change and thus explore participants’ interactions with the programme and their perceptions of *how* it triggered change. A subset of coding was reviewed by the second author (SV). Where disagreements emerged discussion took place until both authors were satisfied. Transcripts were not returned to participants in advance of coding. However, a summary of the results section, including all quotes proposed to be used in the submitted manuscript, was provided to the participants to ensure that the data had been correctly interpreted and the results were an accurate reflection of the discussions. Routinely collected data were analysed descriptively.

### Research team and reflexivity

NM is a physiotherapist by background and has an MA in Health Promotion. She has been working as a research physiotherapist on a predominantly qualitative study which aims to develop a theory-informed behaviour change intervention to change the professional practices of stroke therapy teams, and as such has past experience with the behaviour change frameworks used in this study. NM established a relationship with the Aspire clients and providers through preparatory public and patient involvement work for a doctoral research fellowship. SV is a lecturer in public policy and health and has extensive experience conducting evaluations of community based health improvement interventions. LC is a physiotherapist and a National Institute for Health Research (NIHR) Career Development Fellow with expertise in intervention development and implementation science. SV and LC had no contact with the participants. The research team are fully independent of the third sector organisation. All participants were aware that the research was being conducted to explore how they experienced the different components of the programme to see if it was possible to identify how components supported them in making positive lifestyle changes.

### Ethical approval

This study was approved by the University of Central Lancashire Research Ethics Committee, number STEMH317.

## Results

### Characteristics of sample

All of the Aspire clients (*n* = 12) consented to participate in the discussions and to have their data accessed by the research team. Three focus groups were conducted with 10 individuals (*n*_1_ = 3, *n*_2_ = 3, *n*_3_ = 4). Due to difficulties in finding a mutually convenient time for all participants, one individual opted to take part in a one-to-one interview and another provided input via email.

The characteristics of the Aspire clients, and the groups in which they trained, are shown in Table 1. Pre- and post-measures for the clients are provided to illustrate changes achieved within the group. The cohort consisted of ten female participants and two male participants. The majority of the cohort (*n* = 10) were white British and two participants were South Asian. The age range of clients was 21 years to 53 years. All clients lost 5 % of their body weight or more over the 26 week period. The percentage weight lost ranged from 5.5 to 32.6 % with an average reduction of 18.8 kg. All clients had improved scores on the Patient Health Questionnaire (PHQ-9) and on the Warwick-Edinburgh Mental Well-being scale.

**Table 1:** Characteristics of the Aspire clients including pre- and post-measures

Group	Age group	Weight (kg) (BMI)		% weight loss	PHQ-9^a^		WEMWBS^b^	
		Pre	Post		Pre	Post	Pre	Post
1	50–54	112.1 (35.10)	93.4 (29.25)	16.7	4	0	52	65
	30–34	120.1 (39.26)	80.9 (26.45)	32.6	9	0	46	63
	40–44	131.0 (49.00)	112.3 (42.01)	14.3	7	6	53	58
	40–44	76.8 (28.55)	65.9 (24.50)	14.2	7	0	51	62
2	25–29	160.3 (51.93)	151.5 (49.08)	5.5	10	3	40	50
	19–24	108.9 (39.52)	90.9 (32.99)	16.5	15	1	37	66
	35–39	141.6 (48.77)	117.6 (40.50)	16.9	19	4	33	60
	50–54	79.1 (30.36)	66.5 (25.53)	15.9	21	8	35	50
3	50–54	100.6 (41.50)	90.1 (37.17)	10.4	10	5	45	56
	50–54	108.9 (43.90)	76.9 (31.00)	29.4	16	6	31	57
	45–49	129.8 (49.83)	115.0 (44.15)	11.4	19	4	35	53
	30–34	98.4 (37.36)	80.9 (30.71)	17.8	9	1	40	60

^a^ *PHQ-9* Patient Health Questionnaire-9

^b^ *WEMWBS* Warwick-Edinburgh Mental Well-being Scale

The characteristics of the Aspire providers are shown in Table 2. To ensure that the anonymity of all participants remains intact, participant codes have not been provided in these tables.

**Table 2:** Characteristics of the Aspire providers

Gender	Age	Ethnicity	Training and qualifications	Experience
M	45–49	South Asian	• Qualified youth and community worker • Level 2 gym Instructor	Has worked with the charity for 7 years, previously worked in community development and youth work for ~15 years.
F	30–34	White British	• BSc Hons Dietetics • Registered with the HCPC • Level 2 gym instructor, working towards level 3	Working with the charity for 7 years, previously worked in the NHS in an inpatient and outpatient capacity

### Change in behaviours

The Aspire programme targets behaviours around healthy eating and physical activity. The determinants of these behaviours that emerged from discussions with clients could be organised into four categories (i) intent and commitment, (ii) knowledge and understanding, (iii) beliefs about capabilities and (iv) social and environmental context. For clarity we have structured the results section around these four categories and have summarised participants’ accounts of how Aspire triggered change for each.

### Changes in intent and commitment

When asked about deciding to apply for the weight loss programme, all clients identified that the timing felt right for them and that they were in the right frame of mind to participate.

**Client:** “When this came around last year and one of the girls at work joined it and she said, “Are you coming?” and I just weren’t in right frame of mind, I knew you know. But this year I just thought right yeah, something clicked and I thought right I’m going to do it and that’s how I ended up here really”

Everybody that applies for Aspire is invited to attend a briefing event where they can find out more about the programme. Aspire is limited to a maximum of 12 people per cohort due to practical limitations around time and staff availability. Therefore applicants who identify at the briefing event that they can be flexible throughout the duration of the programme are more likely to be successful. Flexibility in this instance refers to the participants’ availability to attend sessions during working hours and in the evenings (e.g. participants working full-time nine to five jobs committed to using annual leave to attend programme sessions). This competitive selection process, which is quite distinct from typical access routes to weight loss programmes, emerged as an mechanism in fostering commitment to the programme.

**Client:** “I’d heard (name provider 1) and (name provider 2) say how flexible you really need to be and I thought, you know what, if I need to take time off work through annual leave or whatever, then I need to make sure that I am flexible.”

**Provider:** “The selection process is basically people, they have to apply themselves, and it’s all self-referral rather than getting a bunch of people who are like “I’m only here because my GP sent me” and that’s quite a big thing I think for people that are on the programme. They want to do it so they’re more likely to be like “I’m ready, this is my time, I’m going for it now…this is my chance to take.”

Along with fostering commitment to the programme, knowing that they have been given a place on the programme, over other individuals who may have benefited equally from it, generated motivation in clients, particularly at times when they felt that they might not be able to continue.

**Client: “**I felt privileged to get a place and I think those first few weeks when it’s really, it feels really hard, from nothing to three sessions, three hard sessions a week and, and you’re thinking ‘I can’t do this, I can’t do this’, and then the fact that it’s a charity and your taking up £2000 worth of place that somebody else could have had…[…] you think, you know, come on, you’ve got a really good opportunity here, you’re privileged to have got on when other people didn’t who deserved it just as much as you.”

### Changes in knowledge and understanding

All Aspire clients discussed the value of learning about food groups and portion control, and in particular how this made them think differently about their choices.

**Client:** “They talk to you about portion control, which is something I never had before. When we did the session on portion control and we actually did a takeaway and converted it and you saw what you could really have, and you know in your head what you used to have…it really made me think differently”

A number of clients discussed how, prior to the programme, they felt that they were relatively well-informed about healthy eating and were surprised to learn where they were going wrong in previous weight loss efforts. This point was stressed by the programme providers who identified that you cannot make any assumptions about clients’ level of knowledge, particularly around healthy eating habits and portion control.

**Client:** “One of the things (name provider) said to us at the beginning was,” Yes, eat five a day but only two portions of fruit simply because it’s full of sugar” and I’m thinking back to the big bowl of pineapple and tangerine and apple and whatever, grapes and all chucked into it and I’d sit there and actually I’d eaten 8 or 900 calories in just fruit and it was just such an eye opener”

**Provider:** “It was not just telling people how to eat healthy but explaining why that’s healthy and that’s not healthy, if we talk about carbohydrates let’s break it down in layman’s terms. And we’ve learnt now to not take anything for granted because what we think everybody knows – some people haven’t got a clue about – so it’s just breaking everything down the best you can”

Along with direct provision of information from the programme providers, clients also identified how their knowledge developed through learning from one another both through face-to-face interactions within group sessions and through their online private social media group.

**Client:** “I think it, what works with the very big groups is that they generate the conversation between us and therefore that’s how everybody learned, it was like peer learning rather than listening to experts”

### Changes in beliefs about capabilities

Extensive discussion took place in all three focus groups on components of the Aspire programme that impacted on clients’ perceptions of their ability to perform the target behaviours and achieve their goals. Throughout the programme there are a number of milestone challenges set for the clients. These include completing a step climb at a local park, completing a 5 K walk/run and a 10 K walk/run. Clients discussed at length the trepidation they felt when first presented with these challenges but also the sense of achievement they felt having successfully overcome them.

**Client: “**When it’s getting closer and closer and you start training for it, I got really nervous about it…and I’m thinking what if it takes me four hours to do …but everyone’s really supportive and (name provider 1) and (name provider 2) say no…we’re there ‘til tea time if we need to be, we’ll get you round there, you’ll do it!”

**Client:** “It’s just amazed me that actually I can run, that I did that 5 K and that I was training towards a 10 K, I did a 10 K…and as I say, all my life I’ve never done exercise, I’ve always hated exercise, you know I’ve never gone running or anything and now just to know that you can actually, anyone can do it…”

Clients discussed how physically demanding the exercise sessions could be at times and there were contrasting experiences shared by the group on the value they placed on being “pushed to their limits”. For example, some discussed how conquering new challenges every week helped build their confidence about what they could physically achieve. However, a number of clients also identified some frustration at not being in control of the exercise session.

**Client:** “In terms of one-to-one physical sessions, just as I think I’d be at my limitations, I’d have to do another minute or I’d have to do another 30 s or another few reps and for me that was a massive achievement because I thought I was on the very edge of my capabilities but then somehow (name provider) got that bit more from you…”

**Client: “**I did enjoy the gym, don’t get me wrong, but I didn’t like having (name provider) there with a finger like this every two seconds [imitating pressing buttons on the machine], you know what I mean…”

Group support was identified by clients as another important component that positively impacted on their beliefs about their ability to persevere and successfully complete the programme. This support was generated in face-to-face sessions and in particular through the online social media group which allowed the clients to foster relationships and engage in activities outside of the formal sessions.

**Client:** “[Social media site] enabled us all to connect outside of the group sessions, and if one of us, and we’ve all been there and said, “You know what, I’m struggling today” and the support you get…”Does anybody fancy doing something this Sunday morning?”, you couldn’t have got that anywhere else…”

A final component that was identified by a number of the clients was the Aspire alumni night held in week three. Past clients who have maintained the changes they made during the programme attend the large group session and share their reflections and experiences with the group (with and without the providers present).

**Client:** “The former Aspire clients was a good one for me because on about the third week, physically, at that big Monday night I thought I don’t know if I can actually do this because it was really hurting but then they [the alumni] came and I listened to the success that they’d had and that really inspired me, listening to people that had actually gone through and done it so I think that was one of my turning points”

### Changes in social and environmental context

The providers discussed how they have learned from past experience the extent to which clients’ home lives can positively and negatively impact on their experience of the programme. To specifically address this issue they introduced a family night early on in the programme, which clients identified as an important component in fostering support from family and friends.

**Provider:** “So what we did to directly tackle that was said, “OK let’s have a family night to bring in husbands, wives, girlfriends, mums, dads, sisters, whoever, children, to bring them in” (a) so they can see how hard their loved ones are working and (b) say well you need to support this person because they’re doing this…and I make an effort to give everybody my business card and say, you know, if you see him cheating, send me a text! [Laughs]”

**Client:** “It wasn’t until I invited them to the family day that they realised just how hard we all work […] [Her best friend] said to me, “I’m really proud of you for doing it and I’ve got a new found respect for you”, and it was at that point for me, that it was a massive turn for my friends and family so the support came a lot stronger and a lot more of it after that session…”

### Programme overview

Due to the intensive nature of the programme (i.e. three sessions a week for 26 weeks) it is not possible to report in detail clients’ interactions with the full range of components that are delivered during the programme (See Additional file 1: Aspire schedule). In an effort to address this, we have compiled an overview of how the components of Aspire, and their behaviour change techniques, interacted with the determinants of behaviours discussed here (Table 3). This is not an exhaustive list as additional components or behaviour change techniques may have been used in individual sessions that were not identifiable through the programme documentation or that did not emerge in the focus groups and interviews. Definitions for each of the behaviour change techniques presented can be found in the Behaviour Change Technique Taxonomy [16].

**Table 3:** Interaction of Aspire components and their behaviour change techniques with the determinants of behaviours

Determinant of behaviour	Function of intervention components	Underpinning behaviour change techniques	Intervention components and delivery methods^a^
Intent	Establish	• Commitment	• Completion of application form (self-referral) • Attendance at briefing event • Agreement to attend 3 sessions a week over a 26 week period • Competitive selection process • Signing of contracts
Knowledge	Educate	• Instruction on how to perform the behaviours	• Big group sessions • Small group sessions • One-to-one sessions • Communication via text and social media
Beliefs about capabilities	Motivate/ Persuade	• Goal setting • Feedback • Verbal persuasion • Paradoxical instructions • Information on/salience of health consequences • Social comparison • Demonstration of behaviour • Modelling • Anticipated regret	• Milestone challenges • Weight loss targets • Visual feedback (progress graphs) • Verbal feedback (e.g. on food diary) • Physical feedback (e.g. revisiting challenging activities) • Feedback from family • Case studies • Letter writing • Visits from past clients • Social media
	Enable	• Support (practical and emotional) • Exposure • Graded tasks	• Social media and text communication • One-to-one sessions • Small group sessions • Milestone challenges
Social and environmental context	Restructure	• Restructuring the physical environment • Restructuring the social environment • Problem solving • Support (practical)	• One to one sessions • Family night

^a^See Additional file 1 for full schedule of intervention components and activities

## Discussion

There is evidence to suggest that weight loss interventions delivered in primary care settings are ineffective [12] and that patients themselves do not view their weight as a medical issue to be dealt with by their general practitioner (GP) or the health service [20]. As such there is a need to identify effective community based programmes that can support people in making positive behaviour changes and thus lose weight. The aim of our study was to explore the mechanisms of change of a novel weight loss programme provided by a third sector organisation. The percentage weight loss of the programme participants ranged from 5.5 to 32.6 % with an average reduction 18.8 kg over the 26 week period. It is important to note the greater duration and intensity of this programme (i.e. three sessions a week for 26 weeks) relative to traditional funded weight loss interventions which may be an important factor in achieving this level of weight loss. These figures therefore may not be easily generalised to less intensive interventions. For example, average weight loss achieved through participation in Weight Watchers, Slimming World and Rosemary Conley have been found to be 4 to 5 kg at 12 weeks [12] and 4 kg at 1 year [21].

In order for people to benefit from any lifestyle intervention, they must first receive the intervention and, where possible, complete a full “dose”. The issue of attrition rates from lifestyle interventions has been well documented [21–26] and there is a need to identify strategies to maximise adherence. Of the 12 individuals that started as part of the fourth Aspire cohort, 11 completed the 26 week programme and an additional client who joined part-way through also completed the full programme. The Aspire clients identified the competitive selection route into the programme as an important factor in fostering commitment at the outset. This has also been identified by exercise referral professionals, whereby a clear distinction is said to exist between clients that have entered the scheme following referral from a health professional and clients who sought out the programme, where the latter were more likely to adhere to the scheme [27]. It is likely that the competitive selection process results in a highly motivated cohort which will have implications for the weight loss achieved. The extent to which the results of this programme can be compared with outcomes of less intensive interventions that do not have a similar selection process may therefore be limited.

In trials of commercial weight loss programmes participants are provided vouchers by their GP entitling them to a set number of free sessions. Participants in these trials have reported a moral and financial obligation to attend the meetings along with not wanting to “let the doctors down” knowing that they were receiving something that was costly [20]. We have identified a similar finding, whereby Aspire clients were motivated to fully participate in the programme knowing that the programme was run by a charity and that they were taking up an expensive place. The set number of vouchers provided to participants in commercial weight loss trials also served to make explicit the time commitment required and what would be involved. The importance of fully understanding the intervention to which you have been referred has been identified as an important factor for exercise referral schemes. Sometimes referred patients have not had the scheme fully explained to them at the outset and therefore it may not actually have been something in which they would have wanted to take part [27]. Clients of Aspire stressed that as a consequence of the briefing evening they were under no illusion as to what was involved in the programme and the commitment that would be required of them for the full duration. Prospective clients have the opportunity to withdraw their application at this stage, which potentially reduces the likelihood of people who will not adhere to the programme continuing with the process.

Another important mechanism that emerged in the present study was the cohesion of the group, and the support and learning generated by the group for the duration of the programme. Group dynamics in fostering peer support was also identified as a key mechanism by Gray and colleagues when developing the FFIT programme [28]. The male participants identified that the group setting, which included men who they perceived to be similar to themselves, allowed them to share ideas and experiences that helped in their weight loss efforts. A lack of group dynamic and support has also been proposed as one potential explanation for the limited effectiveness of primary care based weight loss programmes when compared to commercial programmes [12]. Although the extent to which individuals attending commercial programmes are facilitated to interact with each other and foster supportive relationships is unclear. Some exercise referral specialists have identified explicitly fostering interaction during exercise classes as a key responsibility to support the emergence of social networks which may last beyond the programme and thus maintain adherence to exercise [27]. A novel component of Aspire that acted as an adjunct to the face-to-face sessions and further generated peer support was the use of a private online social media group. The use of social media as part of the programme is something that has evolved over time and there are a now a number of subgroups that clients can join, e.g. for smaller training groups or alumni. The latter represents a “community” of people that have participated in Aspire and acts as a means of facilitating ongoing contact between clients and providers. There is at present a paucity of research on the value of social media as a component of weight loss interventions and further research is needed to demonstrate its role and effectiveness [29].

As the explicit purpose of the focus group discussions was to explore clients’ interactions with the components of Aspire, and the ways in which these components supported them to make a change, there was very little discussion on clients’ weight loss history and their own personal feelings about their weight. However, it was evident that clients were surprised, and at times overwhelmed, by their physical achievements during the programme and that perhaps as a consequence of their weight had not believed that such achievements were possible for them. The experience of not achieving desired weight loss has been found to result in a “blaming” discourse amongst people who are overweight or obese that can negatively impact on self-esteem [30, 31]. In previous weight loss studies, behaviour change techniques such as self-monitoring [32], focusing on past successes and verbal persuasion about capability [28] have been employed to try and generate self-belief in participants. The Aspire providers are completely autonomous in how they choose to run the programme and the activities that they deliver. As a result, they are able to go beyond “traditional” methods or techniques to foster self-belief and employ more dynamic and novel strategies such as group participation in local 5 K and 10 K races. The providers also use milestone challenges (e.g. a step climb at a local park) at the beginning and end of the programme to demonstrate to participants how much they have achieved over the 26 weeks. It was evident that these milestones were stand-out moments in the programme for clients and also acted as a means to further establish relationships and a supportive environment within the group.

Changes in social and environmental context represent a final mechanism that merits discussion, again due to the novel and targeted nature of the component used to bring about the change. There is a wealth of literature demonstrating the value and effectiveness of including social support in weight loss interventions but limited examples as to how this can be optimally achieved [33]. Families are most often discussed in the weight loss literature as the driving force for change, in the sense that participants are aware that their weight is negatively impacting on their family life, or on their health, which may shorten the time they have with their family [30]. The inclusion of a family night early on in the Aspire programme was identified as an important turning point for a number of participants in ensuring that their family members understood the level of commitment they were making to the programme and how hard they were working at each of the sessions. Family members were not interviewed as part of this study but it is possible that there may have been a “knock on” effect whereby family members became more engaged in health eating and being more active as a consequence of their exposure to the programme.

### Strengths and limitations

One of the main limitations of primary research on the effectiveness of weight loss interventions is a failure to pay sufficient attention to the underlying theory as to how interventions are proposed to bring about change. A strength of this study is the use of established behaviour change theory in the form of the Theoretical Domains Framework, the Behaviour Change Wheel and the Behaviour Change Technique Taxonomy to guide data collection and analysis. This has ensured a systematic approach to examining the weight loss programme and use of consistent terminology for future comparisons.

All clients of the fourth cohort participated in data collection and member checking in some way and therefore we are confident that all potentially relevant experiences and perspectives have been captured. However as all participants were from a single cohort, a group effect may have been present resulting in polarising of discussions. The experiences of the relatively small number of clients included in this study are specific to the programme under investigation. However, by focusing on mechanisms of change and positioning these findings in context with the current evidence base we believe we have increased the transferability of the findings. Clients were aware that the programme providers would have an opportunity to see anonymised quotes during member checking and in the final publication and this may have resulted in social desirability bias, whereby clients were more positive about the programme. However, every effort was made to ensure that it would not be possible to identify the clients and thus encourage candid accounts of their experiences.

## Conclusion

There is an urgent need to establish robust interventions that can support people who are overweight and obese to achieve a healthy weight and maintain this change. We have presented findings from one example of a novel community-based weight loss programme, highlighted some of the core components of this programme (e.g. competitive self-referral access route, group and online support, family involvement and overcoming milestone challenges) and identified how they have resulted in change amongst the participants. However, as with all interventions there is no “one size fits all” approach. Despite recent evidence demonstrating the effectiveness of commercial weight loss programmes, the dropout rate from these interventions remains unacceptably high and there is a need for further research to build up an evidence base on what types of weight loss interventions work for whom, how and under what circumstances to effectively overcome this challenge. Attrition rates for this particular programme are unusually low and therefore may provide a feasible and effective alternative for individuals that have struggled to achieve their desired weight loss through public or private sector programmes. However further research is needed to robustly test the effectiveness, and cost-effectiveness, of the programme.

## Ethics

All participants provided written informed consent. This study was approved by the University of Central Lancashire Research Ethics Committee, number STEMH317.

## Consent to publish

Not applicable.

## Availability of data and materials

The interview data is not available for sharing as consent to share or archive was not obtained from the participants in this study.

## Additional files

Additional file 1: Aspire schedule. Content of the 26 weekly sessions. (PDF 26 kb) Additional file 2: COREQ checklist. Consolidated criteria for reporting qualitative research checklist. (PDF 144 kb) Additional file 3: Topic guides. Topic guides used for the focus groups and interviews. (PDF 94 kb)

## Abbreviations

BMI: body mass index
FFIT: Football Fans in Training
NHS: National Health Service
NICE: National Institute for Clinical Excellence
NIHR: National Institute for Health Research
PHQ-9: Patient Health Questionnaire
STEMH: Science, Technology, Engineering, Medicine and Health
UK: United Kingdom
