# Supplementary material for: Mechanisms of change of a novel weight loss programme provided by a third sector organisation: a qualitative interview study
Source: BMC Public Health. 2016 May 10;16:378. doi: 10.1186/s12889-016-3063-4 (PMC4862220; doi:10.1186/s12889-016-3063-4)
Supplement: Additional file 1: — Aspire schedule. Content of the 26 weekly sessions. (PDF 26 kb) [file 12889_2016_3063_MOESM1_ESM.pdf]

Additional file 1: Aspire schedule

Throughout Aspire there is a weekly ‘big group session’, ‘small group exercise session’ and ‘1-to-1 session’. ‘Big group sessions’ consisted of an hour discussion (detailed below) and a circuit training session with progressive introduction of different pieces of equipment. Varying set-ups each week included simple circuits, timed exercises, paired workouts, games using dice, army drill circuit, outdoor training. Small group exercise sessions involved hour long physical activity in groups of four. 1-to1 sessions consisted of discussion around individual issues and physical activity. Additional activities provided during the programme are also detailed below.

Week	Big group discussion	Other activities
**1**	• Signing ‘contracts’, recap of 6 months, give out plates, bowls and food diaries.<br>• Weight and body fat measurements<br>• Mental/emotional health questionnaires (PHQ-9 and Warwick-Edinburgh)	• Saturday – induction: diet plans, exercise/ circuit training technique, sessions for increasing food knowledge<br>• Sunday – fitness assessments and initial waist, hip and chest measurements done in small groups
**2**	• Working out ‘portions’ from food labels	
**3**	• Meal planning<br>• Hunger vs craving<br>• Triggers to over eating	
**4**	• First visit from previous Aspire clients	
**5**	• Case study on a group leaders weight loss experience	
**6**	• Drinks (alcoholic and non-alcoholic)	
**7**	• Cooking – healthy lunches, focus on lunch away from home	
**8**	• Family exercise session	
**9**	• Write encouraging letter to yourself (these are returned to participants later on in the programme)<br>• Discussion around the hardest/ easiest things so far	

**10**	• Takeaways	• Optional group walk (Saturday), friends and family also invited
**11**	• Cooking – evening meal ideas<br>• Weight loss graphs	
**12**	• Second visit from previous Aspire clients	• Mid-way fitness assessments, weight, body composition, measurements
**13**	• 5k challenge preparation	
**14***	• Halfway questions – what has been most helpful, what else would they like to know – ranked importance by group	• Saturday 5k challenge
**15**	• Mental health and weight loss	
**16**	• Giving out and sharing letters written in week 9	
**17**	• Receiving and accepting compliments	
**18**	• Principles of exercise and how to plan a workout	• Optional group walk (Saturday), friends and family also invited
**19**	• Dealing with celebrations (focus on Christmas and New Year or Easter and bank holidays depending on time of year)	
**20**	• Continuation from week 19	
**21**	• Question and answer session	
**22**	• What happens after Aspire	
**23**	• Previous Aspire written case studies/ reasons given for weight regain after finishing Aspire	
**24**	• Graph showing individuals BMI change, with discussion around health risks still associated with being at that BMI	• Saturday 10k challenge
**25**	• Bring and show old clothing from the beginning of Aspire	
**26**	• Personal journey packs	

*Depending on what is discussed on week 14, subsequent sessions are subject to change
