# Supplementary material for: Mechanisms of change of a novel weight loss programme provided by a third sector organisation: a qualitative interview study
Source: BMC Public Health. 2016 May 10;16:378. doi: 10.1186/s12889-016-3063-4 (PMC4862220; doi:10.1186/s12889-016-3063-4)
Supplement: Additional file 2: — COREQ checklist. Consolidated criteria for reporting qualitative research checklist. (PDF 144 kb) [file 12889_2016_3063_MOESM2_ESM.pdf]

Additional file 2: Consolidated criteria for reporting qualitative studies (COREQ): 32-item checklist

Developed from:
Tong A, Sainsbury P, Craig J. Consolidated criteria for reporting qualitative research (COREQ): a 32-item checklist for interviews and focus groups. *International Journal for Quality in Health Care*. 2007. Volume 19, Number 6: pp. 349 – 357

No. Item	Guide questions/description	Reported in section:
**Domain 1: Research team and reflexivity**		
Personal Characteristics		
1. Inter viewer/facilitator	Which author/s conducted the inter view or focus group?	Data collection
2. Credentials	What were the researcher's credentials? E.g. PhD, MD	Research team and reflexivity
3. Occupation	What was their occupation at the time of the study?	Research team and reflexivity
4. Gender	Was the researcher male or female?	Research team and reflexivity
5. Experience and training	What experience or training did the researcher have?	Research team and reflexivity
Relationship with participants		
6. Relationship established	Was a relationship established prior to study commencement?	Research team and reflexivity
7. Participant knowledge of the interviewer	What did the participants know about the researcher? e.g. personal goals, reasons for doing the research	Research team and reflexivity
8. Interviewer characteristics	What characteristics were reported about the inter viewer/facilitator? e.g. Bias, assumptions, reasons and interests in the research topic	Research team and reflexivity
**Domain 2: study design**		
Theoretical framework		
9. Methodological orientation and Theory	What methodological orientation was stated to underpin the study? e.g. grounded theory, discourse analysis, ethnography, phenomenology, content analysis	Data analysis
Participant selection		
10. Sampling	How were participants selected? e.g. purposive, convenience, consecutive, snowball	Sample
11. Method of approach	How were participants approached? e.g. face-to-face, telephone, mail, email	Sample
12. Sample size	How many participants were in the study?	Sample
13. Non-participation	How many people refused to participate or dropped out? Reasons?	Characteristics of sample

Setting		
14. Setting of data collection	Where was the data collected? e.g. home, clinic, workplace	Study design and setting
15. Presence of non-participants	Was anyone else present besides the participants and researchers?	Data collection
16. Description of sample	What are the important characteristics of the sample? e.g. demographic data, date	Characteristics of the sample
Data collection		
17. Interview guide	Were questions, prompts, guides provided by the authors? Was it pilot tested?	Data collection
18. Repeat interviews	Were repeat inter views carried out? If yes, how many?	Data collection
19. Audio/visual recording	Did the research use audio or visual recording to collect the data?	Data collection
20. Field notes	Were field notes made during and/or after the inter view or focus group?	Data collection
21. Duration	What was the duration of the inter views or focus group?	Data collection
22. Data saturation	Was data saturation discussed?	Data collection
23. Transcripts returned	Were transcripts returned to participants for comment and/or correction?	Data analysis
**Domain 3: analysis and findings**		
Data analysis		
24. Number of data coders	How many data coders coded the data?	Data analysis
25. Description of the coding tree	Did authors provide a description of the coding tree?	Data analysis
26. Derivation of themes	Were themes identified in advance or derived from the data?	Data analysis
27. Software	What software, if applicable, was used to manage the data?	Data analysis
28. Participant checking	Did participants provide feedback on the findings?	Data analysis
Reporting		
29. Quotations presented	Were participant quotations presented to illustrate the themes/findings? Was each quotation identified? e.g. participant number	Results
30. Data and findings consistent	Was there consistency between the data presented and the findings?	Results
31. Clarity of major themes	Were major themes clearly presented in the findings?	Results
32. Clarity of minor themes	Is there a description of diverse cases or discussion of minor themes?	Results
