# Supplementary material for: Mechanisms of change of a novel weight loss programme provided by a third sector organisation: a qualitative interview study
Source: BMC Public Health. 2016 May 10;16:378. doi: 10.1186/s12889-016-3063-4 (PMC4862220; doi:10.1186/s12889-016-3063-4)
Supplement: Additional file 3: — Topic guides. Topic guides used for the focus groups and interviews. (PDF 94 kb) [file 12889_2016_3063_MOESM3_ESM.pdf]

Additional file 3: Topic guides

Topic guide for focus group with Aspire participants

**Introductions**
• How did you make the decision to apply for the programme?<br>• What were your experiences of the application process?
**Getting started with the programme**
• Did you have any reservations on starting the programme?<br>• How did you find getting going at the start?<br>• Was there anything that helped or got in the way of getting going?
**Experiences of the programme**
• What changes have you made in your life as a result of participating in the programme to help you lose weight/maintain weight loss?<br>• What aspects of the programme do you think were most effective for you in making these changes? What was it about these…(e.g. education, exercise, 1-to-1 sessions, Facebook)<br>• Were there aspects of the programme that you think didn't work as well for you?
**Reflections on the programme**
• What has been the biggest impact that participating in the programme has had on your life?<br>• If the programme was to be delivered by other organisations, what would be the most important qualities of the providers?<br>• If you could do it all again, is there anything you would change for yourself?
**Future**
• How has the transition from being "on" the programme to being "off" the programme been?<br>• Going forwards, how are you feeling about maintaining your weight loss?

Topic guide for semi-structured interviews with Aspire providers

Introductions and development of the programme
• Can you talk to me a little bit about the history of the programme and how it came to exist e.g. funding, development?<br>• Were there existing programmes on which it was modelled?<br>• What were the main objectives (or target behaviours)?<br>• How did you envisage that the programme would result in changing these behaviours?
**Selection process**
• Can you describe the selection process, and how this came about?<br>• How do you select participants (e.g. distinguishing characteristics)?<br>• From the people who start the programme, how many complete it?<br>• For people who have dropped out in the first few weeks, do you know the reasons for this and do you think this could have been pre-empted?
**The programme**
• Can you describe how the various components came about and who was responsible for their development?<br>• At the outset did you have in mind how each of the components could tackle certain issues around weight loss behaviours?<br>• From your experience of the programme, what is it about the programme that results in people making a change and losing weight?<br>• How have you modified the programme over the four cohorts?<br>• How consuming is the programme for you personally?<br>• Are you ever concerned that individuals will become reliant on the programme or the support of the providers?<br>• Are there certain types of people that thrive more on programme than others?<br>• To what extent do people continue with lifestyle change after the programme to achieve their target weight loss?
**Continuation**
• What plans are in place for continuing to provide the programme?<br>• Are there plans to make further modifications to the programme going forwards?<br>• Blue sky thinking, what would you like to do with the programme? What are the principles that underpin the success of the programme?
